# Honey isomaltose contributes to the induction of granulocyte-colony stimulating factor (G-CSF) secretion in the intestinal epithelial cells following honey heating

**DOI:** 10.1038/s41598-020-71993-w

**Published:** 2020-09-16

**Authors:** Xin Xu, Koshi Asai, Daiki Kato, Kan’ichiro Ishiuchi, Kewen Ding, Yoshiaki Tabuchi, Misato Ota, Toshiaki Makino

**Affiliations:** 1 Department of Pharmacognosy, Graduate School of Pharmaceutical Sciences, Nagoya City University, 3-1 Tanabe-Dori, Mizuho-ku, Nagoya, 467-8603 Japan; 2 Kuki Sangyo Co. Ltd., 11 Onoe-cho, Yokkaichi, Mie 510-0059 Japan; 3 Division of Molecular Genetics Research, Life Science Research Center, Toyama University, 2630, Sugitani, Toyama, 930-0194 Japan

**Keywords:** Drug development, Pharmacodynamics, Monosaccharides, Natural product synthesis

## Abstract

We have previously discovered that heated honey but not unheated honey could induce the secretion of granulocyte-colony stimulating factor (G-CSF) in the MCE301 intestinal epithelial cells. The objective of this study was to identify compounds in honey that could contribute to this activity. We bought several kinds of commercial honey samples derived from different flowers, as well as corn syrup samples, in the markets of China and Japan, and heated them at 180 °C for 30 min. MCE301 cells were treated with the medium containing the samples, and G-CSF levels in the medium were measured by ELISA. By comparing their activities and sugar contents, we discovered that isomaltose was primarily implicated. The optimum heating conditions for isomaltose were at 180 °C for 60 min or at 200 °C for 15–30 min, and these time- and temperature-dependencies were similar to those of honey in our previous study. When heated isomaltose was partitioned by dialysis, the active ingredients were transferred into a high-molecular-weight fraction. By size-exclusion HPLC analysis, the average molecular weight of heated isomaltose was 790 kDa. When heated isomaltose was hydrolyzed by acids, glucose was subsequently produced. Maltose, sucrose, turanose, and trehalose did not exhibited any activity when heated at 180 °C for 60 min, indicating that the glucose groups with *α*(1 → 6)-binding in the isomaltose molecule play important roles in its activity when oxidatively polymerized by heat. The stimulating activity of heated isomaltose was inhibited by toll-like receptor 4 (TLR4) inhibitor, suggesting that heated isomaltose activates TLR4 to induce G-CSF. Since G-CSF is clinically used for cancer patients to accelerate their recovery from neutropenia following chemotherapy or accompanied with aplastic anemia, these findings indicate that honey which contains high level of isomaltose could improve immunosuppressive conditions when honey is heated, and that heated isomaltose might be of potential therapeutic use in patients with compromised immunity caused by chemotherapeutic agents.

## Introduction

Honey is a sweet food that has been consumed since ancient times, originating from nectar and honeydew gathered from various flowers by bees and transformed into a viscous substance^1,2^. Notably, traditional Chinese medicine (TCM) uses it as a pharmaceutical additive in the preparation of crude drugs derived from medicinal plants. The Chinese Pharmacopoeia^3^ described “stir-baking with honey” as the preparation method of crude drugs.

Previously, we discovered that heat-processed honey but not unheated honey could induce the secretion of granulocyte-colony stimulating factor (G-CSF) in the MCE301 intestinal epithelial cells^4^. G-CSF is a cytokine that promotes the proliferation, differentiation, and maturation of granulocytes^5,6^ and regulates hematopoiesis and innate/adaptive immunity^7,8^. G-CSF is clinically used for certain cancer patients to accelerate their recovery from neutropenia following chemotherapy or accompanied with aplastic anemia^9^, and to prevent from peripheral neuropathy as the adverse effect of anti-cancer drugs^10^, suggesting that heated honey might improve reduced immune function, anemia, and neuropathy by chemotherapeutic agents or other stresses. It is theorized in TCM that the aim of “stir-baking with honey” processing is tonifying *qi* (vital energy) and strengthening the immune function in the intestine^11,12^. Our previous findings revealed that licorice with “stir-baking with honey” processing heated at 180 °C for 1 h actuated G-CSF release^13^. Moreover, the active constituents of heated honey had an average molecular weight of 730 kDa, and trifluoroacetic acid (TFA) hydrolyzation induced galactose, glucose, 5-(hydroxymethyl)-furfural (5-HMF), and *α*-ribofuranose *β*-ribofuranose 1,5′:1′,5-dianhydride released from them^4^.

For the present study, our objective was to identify the components in honey that could induce the heating-triggered secretion of G-CSF in MCE301 cells. Since the type and concentration of sugars, phenolic acids, and flavonoids in honey vary depending on the plant species of origin and the collection places^14,15^, multiple honey samples were examined in order to better understand how these factors could affect the differences in G-CSF secretion. The results of this evaluation would enable the establishment of the criteria for honey suitable for an application in TCM.

## Results

### The effect of honey and corn syrup samples, with or without heat-processing, on the G-CSF secretion induction in MCE301 cells

**Figure 1:**
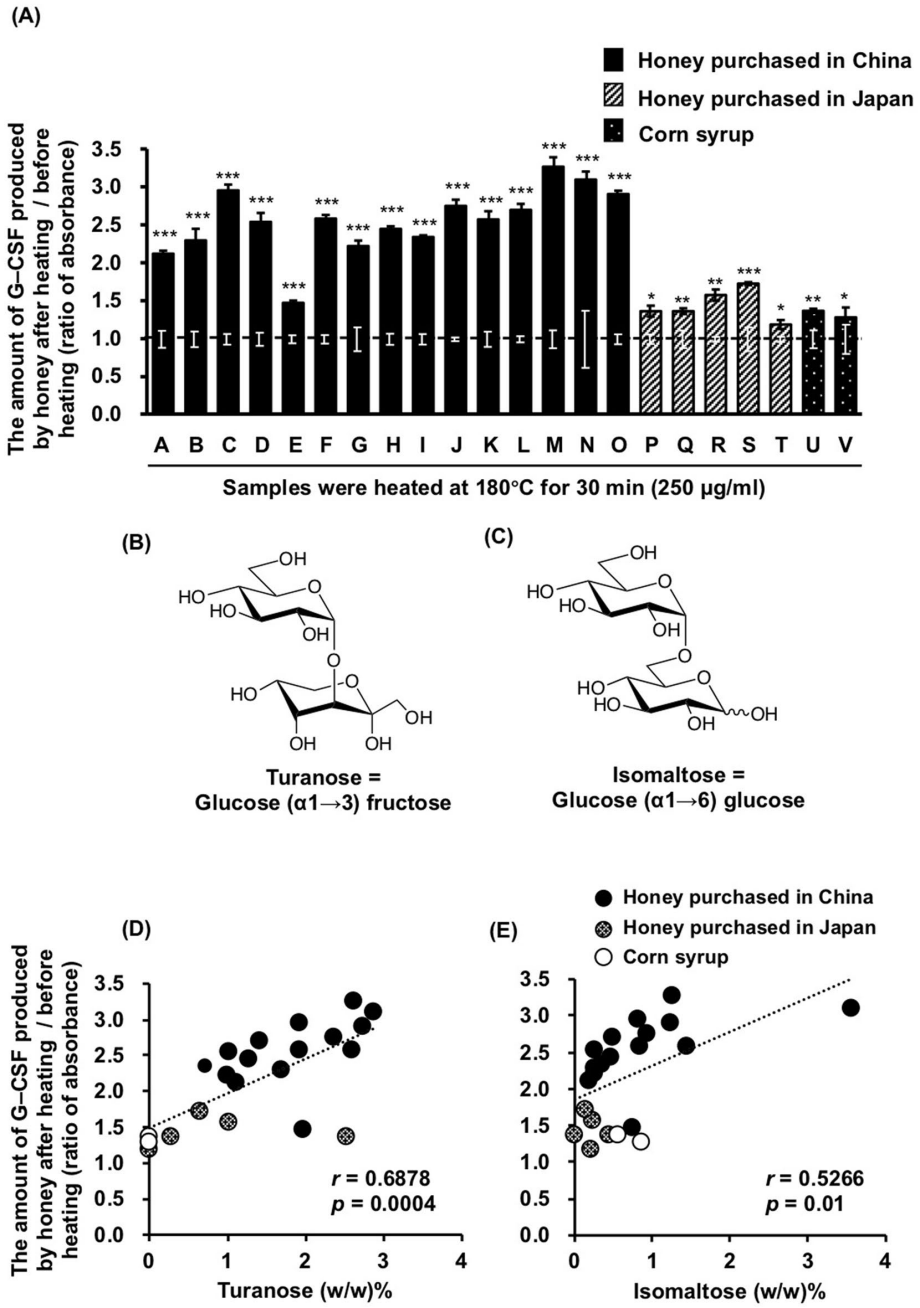
Differences in the inducible effect of honey and its related products, purchased in China and Japan, on G-CSF secretion in MCE301 cells. (**A**) Each sample was either heated to 180 °C for 30 min or unheated, then dissolved in a medium at 250 µg/ml. Cells were incubated for 24 h and the G-CSF levels in the medium were measured by ELISA. Data are shown as the ratio of the heated sample absorbance and that of the unheated sample. The letters shown on X-axis indicates the sample codes shown in Table 1. *p < 0.05, **p < 0.01, ***p < 0.001 between the unheated honey group and the heated honey group shown with the same letters for Student’s *t*-test. Data are represented as the mean ± S.E. (*n* = 3). (**B**,**C**) The structures of turanose and isomaltose, respectively. (**D**,**E**) Scatter plot between the inducible effects on the G-CSF secretion in MCE301 cells and the amounts of turanose and isomaltose in the honey samples. The correlation coefficient was calculated using the Pearson correlation analysis and regression analyses. The Y-axis shows the increased activity (**A**, **D**, **E**). 0.4 < *r* < 0.7 indicates a positive correlation between the two groups of data.

We purchased twenty honey and two corn syrup samples from companies in China and Japan in 2012 and 2017 (Table 1). The G-CSF secretion in the MCE301 cells was significantly enhanced in the cells treated with samples heated at 180 °C for 30 min, compared to cells treated with unheated samples. Interestingly, honey purchased in China tended to show a stronger enhancement than honey purchased in Japan or corn syrup samples (Fig. 1A).

**Table 1:** List of honey samples and their corresponding extraction ratios.

Sample code	Samples		Company name purchased	Lot number	Ratio (w/w) % of the extract yielded to unheated one	Ratio (w/w) % of the weight of heated sample to unheated one
A	Honey purchased in China	Acacia^a^	Chinese Academy of Apicultural Sciences, 2017^o^	16CD2038	89.2	70.8
B		Acacia mature^b^	China Oil & Foodstuffs Industry, 2017^p^	HY1211029X	89.9	76.6
C		Astragalus^c^	Beijing Tongrentang Health Pharmaceutical, 2012^q^	1102005	88.7	69.3
D		Leonurus^d^	Beijing Tongrentang Health Pharmaceutical, 2012^q^	1102005	86.6	70.2
E		Leonurus^d^	Shanghai Hujiao Bee Industry Association, 2017^r^	–	88.8	74.8
F		Longan^e^	Beijing Tongrentang Health Pharmaceutical, 2012^q^	1102005	86.2	71.0
G		*Tilia miqueliana*^f^	Beijing Tongrentang Health Pharmaceutical, 2012^q^	1102004	87.0	70.6
H		Lycium^g^	Beijing Tongrentang Health Pharmaceutical, 2012^q^	1102004	87.3	69.5
I		Chinese milk vetch^h^	Beijing Tongrentang Health Pharmaceutical, 2012^q^	1102004	89.2	71.5
J		Sunflower^i^	Beijing Tongrentang Health Pharmaceutical, 2012^q^	1102004	87.7	70.0
K		Jujube^j^	Chinese Academy of Apicultural Sciences, 2017^o^	16CD3023	88.5	66.1
L		Jujube^j^	Beijing Baihuafengye Technology Development, 2017^s^	20160701A3	86.2	68.2
M		Jujube^j^	Shanghai Hujiao Bee Industry Association, 2017^r^	–	86.1	67.7
N		Jujube^j^	Beijing Tongrentang Health Pharmaceutical, 2012^q^	1102005	88.9	65.4
O		Jujube mature^k^	China Oil & Foodstuffs Industry, 2017^p^	HZ1611090Y	87.6	69.0
P	Honey purchased in Japan	Unknown^l^	Nihon Yoho, 2017^t^	160421	89.9	73.8
Q		Acacia^a^	Api, 2017^u^	–	88.2	71.9
R		Chinese milk vetch^h^	Api, 2017^u^	–	87.5	73.0
S		Buckwheat^m^	Api, 2017^u^	901051	86.6	70.8
T		Unknown^n^	Api, 2017^u^	138061	91.9	73.8
U	Corn syrup	High fructose	Api, 2017^u^	17.4.11	84.6	68.2
V		High glucose	Api, 2017^u^	28.9.07	80.8	65.8

Sample codes are given. Honey samples were defined as the origin of honey purchased, their source of flower names, and lot numbers. Corn syrup samples were defined as the company names and lot numbers. The ratio (w/w) % of the extract yielded the unheated sample and the ratio of the weight of the heated sample to that of the unheated sample was measured as descried in the Methods section.

^a^Flower of *Robinia pseudoacaci*a.

^b^Matured flower of *Robinia pseudoacaci*.

^c^Flower of *Astragalus membranaceus*.

^d^Flower of *Leonurus japonicus*.

^e^Flower of *Dimocarpus longan*.

^f^Flower of *Tilia miqueliana.*

^g^Flower of *Lycium barbarum*.

^h^Flower of *Astragalus sinicus*.

^i^Flower of *Helianthus annuus*.

^j^Flower of *Ziziphus jujube*.

^k^Matured flower of* Ziziphus jujube*.

^l^Source of flower is unknown, but the sample was standardized by Japanese Pharmacopoeia VII Edition.

^m^Flower of *Fagopyrum esculentum*.

^n^Source of flower was unknown but the name of product is "refined honey".

^o^Institute of Apicultural Research, Chinese Academy of Apicultural Sciences, Beijing, P.R.China.

^p^China Oil & Foodstuffs Industry, Beijing, P.R.China.

^q^Beijing Tongrentang Health Pharmaceutical, Beijing, China.

^r^Shanghai Hujiao Bee Industry Association, Shanghai, P.R.China.

^s^Beijing Baihuafengye Technology Development, Beijing, P.R.China.

^t^Nihon Yoho, Gifu, Japan.

^u^Api, Gifu, Japan.

### Sugar contents in honey and corn syrup products

The sugar contents in honey and corn syrup products were measured by high-performance liquid chromatography (HPLC) (Table 2), and the representative chromatogram is shown in Supplementary Fig. 1. Fructose and glucose were the major sugars in the samples, comprising more than 40% and 30% of their makeup, respectively. Sucrose, turanose, maltose, and isomaltose were detected in minor amounts. Although earlier studies reported the presence of trehalose and melezitose in certain honey samples^16–18^, our study detected neither. The glucose content in honey samples purchased in China (46.3 ± 3.4%) was significantly lower than that in Japan (49.0 ± 3.1%, *p* < 0.05, Student’s *t*-test). However, we could not observe any statistically significant difference between the honey samples purchased in China and Japan among the contents of other sugars and the glucose/fructose (G/F) ratio.

**Table 2:** The contents (w/w)% of each observed sugar and the ratio of glucose and fructose (G/F) in the samples.

Sample code	Fructose	Glucose	Sucrose	Turanose	Maltose	Isomaltose	Total^a^	G/F ratio^b^
A	46.0	36.3	0.3	1.1	1.7	0.2	85.6	1.3
B	46.9	33.6	0.2	1.7	1.6	0.3	84.3	1.4
C	41.9	35.8	0.1	1.9	1.5	0.8	82.0	1.2
D	51.5	37.4	0.2	1.0	1.3	0.3	91.7	1.4
E	40.9	36.8	0.1	2.0	1.7	0.7	82.3	1.1
F	40.3	38.7	0.1	1.9	2.4	0.8	84.3	1.0
G	47.2	37.1	n.d.	1.0	1.4	0.3	86.9	1.3
H	45.9	35.7	0.2	1.3	1.6	0.5	85.2	1.3
I	47.5	34.1	0.1	0.7	1.9	0.4	84.7	1.4
J	45.1	38.1	0.1	2.4	1.5	0.9	88.1	1.2
K	42.0	32.6	n.d.	2.6	1.2	1.4	79.9	1.3
L	40.2	39.1	n.d.	1.4	1.1	0.5	82.4	1.0
M	42.4	37.2	n.d.	2.6	2.0	1.3	85.5	1.1
N	40.7	25.9	0.1	2.9	1.1	3.6	74.3	1.6
O	46.5	36.6	n.d.	2.7	2.0	1.2	89.2	1.3
P	51.6	38.0	0.1	0.3	0.7	n.d.	90.7	1.4
Q	50.5	34.4	0.2	2.5	1.8	0.4	89.9	1.5
R	46.5	32.8	0.2	1.0	1.8	0.2	82.5	1.4
S	51.5	33.5	0.2	0.7	1.0	0.1	86.9	1.5
T	44.8	35.3	0.2	n.d.	1.0	0.2	81.5	1.3
U	49.4	38.7	0.1	n.d.	1.2	0.6	90.0	1.3
V	43.1	45.4	n.d.	n.d.	1.5	0.9	90.9	0.9

The contents of each sugar in each dried sample was measured as described in Materials section and expressed as (w/w) %. The n.d. means less than 0.05 %, and trehalose and melezitose in all the samples were n.d. Total^a^ is the sum of each sugar shown to the dried weight of the sample. The G/F ratio^b^ is the ratio of the content of glucose content to that of fructose.

### The effect of heated glucose and fructose on inducing G-CSF secretion in the MCE301 cells

**Figure 2:**
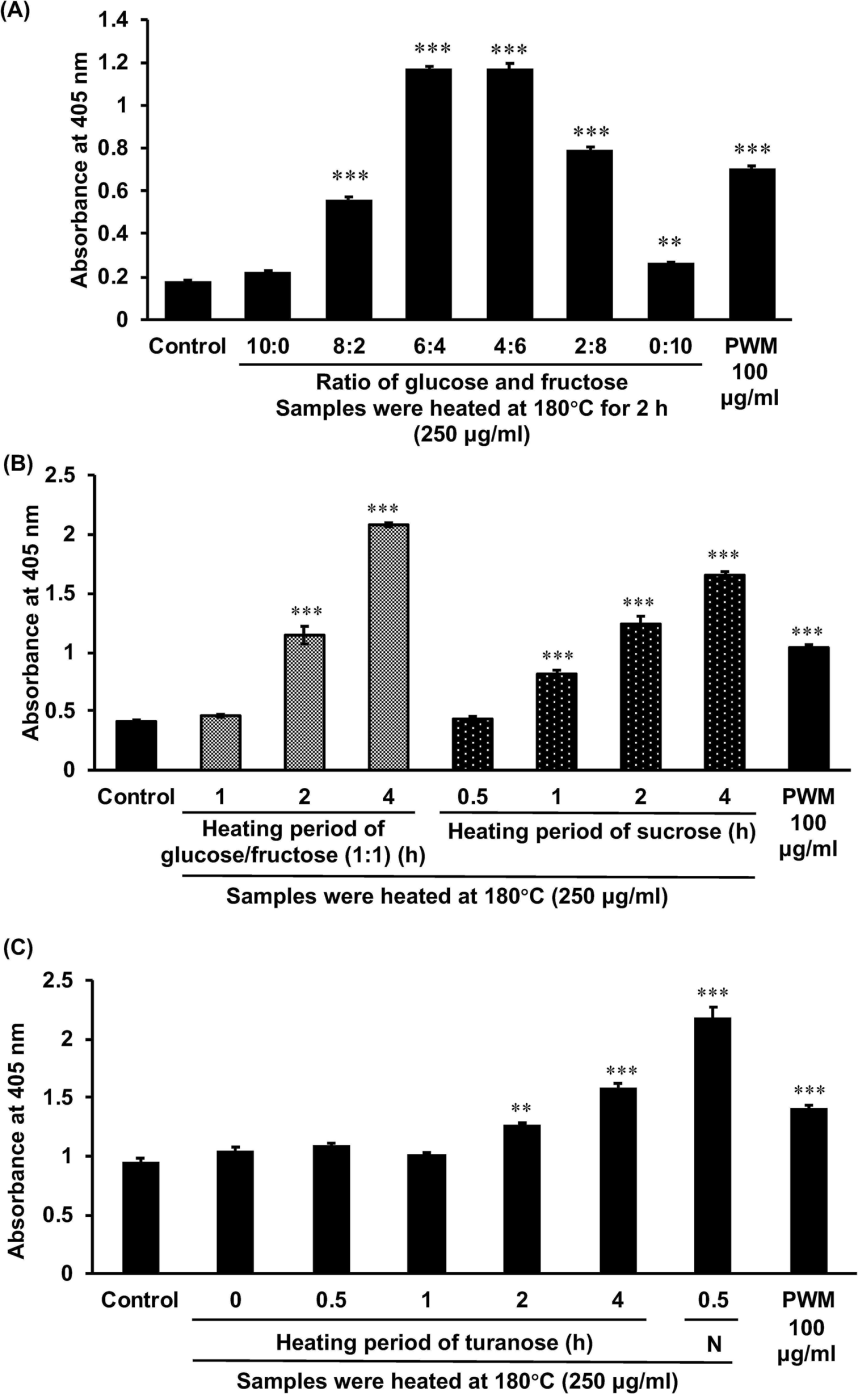
Inducible effect of heated sugar samples on G-CSF secretion from MCE301 cells. (**A**) The mixture of glucose and fructose, ratio is shown on the X-axis, heated at 180 °C for 2 h. (**B**) The mixture of glucose and fructose (1:1) or sucrose was heated at 180 °C for the duration shown on the X-axis. (**C**) Turanose or honey sample N were heated at 180 °C for the duration shown on the X-axis. After being heated, the samples were dissolved in the medium at 250 µg/ml, and cells were incubated for 24 h. The G-CSF levels in the medium were measured by ELISA and the data were shown as the absorbance. **p < 0.01 and ***p < 0.001 against the control group by Dunnet’s and Dunn’s multiple *t*-tests. Data are represented as the mean ± S.E. (*n* = 6). *PWM* pokeweed mitogen.

When glucose and fructose were heated at 180 °C for 2 h, heated fructose exhibited a slight but significant inducible effect but heated glucose did not. When these sugars were mixed, the inducible effect increased, and the titer became the highest when the ratio of the mixture was around 1:1 (Fig. 2A). The effect appeared to be dependent on the heating duration, as heating it for 1 h did not result in the induction of G-CSF secretion, while heating it for 2 h led to a modest, and 4 h to a significantly enhanced induction. The effect of the glucose/fructose (1:1) mixture was similar to that of sucrose (Fig. 2B).

### The correlation between the sugar contents and G-CSF secretion induction

Of all the sugars examined, the contents of turanose (Fig. 1B,D) and isomaltose (Fig. 1C,E) in the honey samples were significantly correlated positively with the induction of the G-CSF secretion when heated.

### Screening for sugars that induce G-CSF secretion upon heating

**Figure 3:**
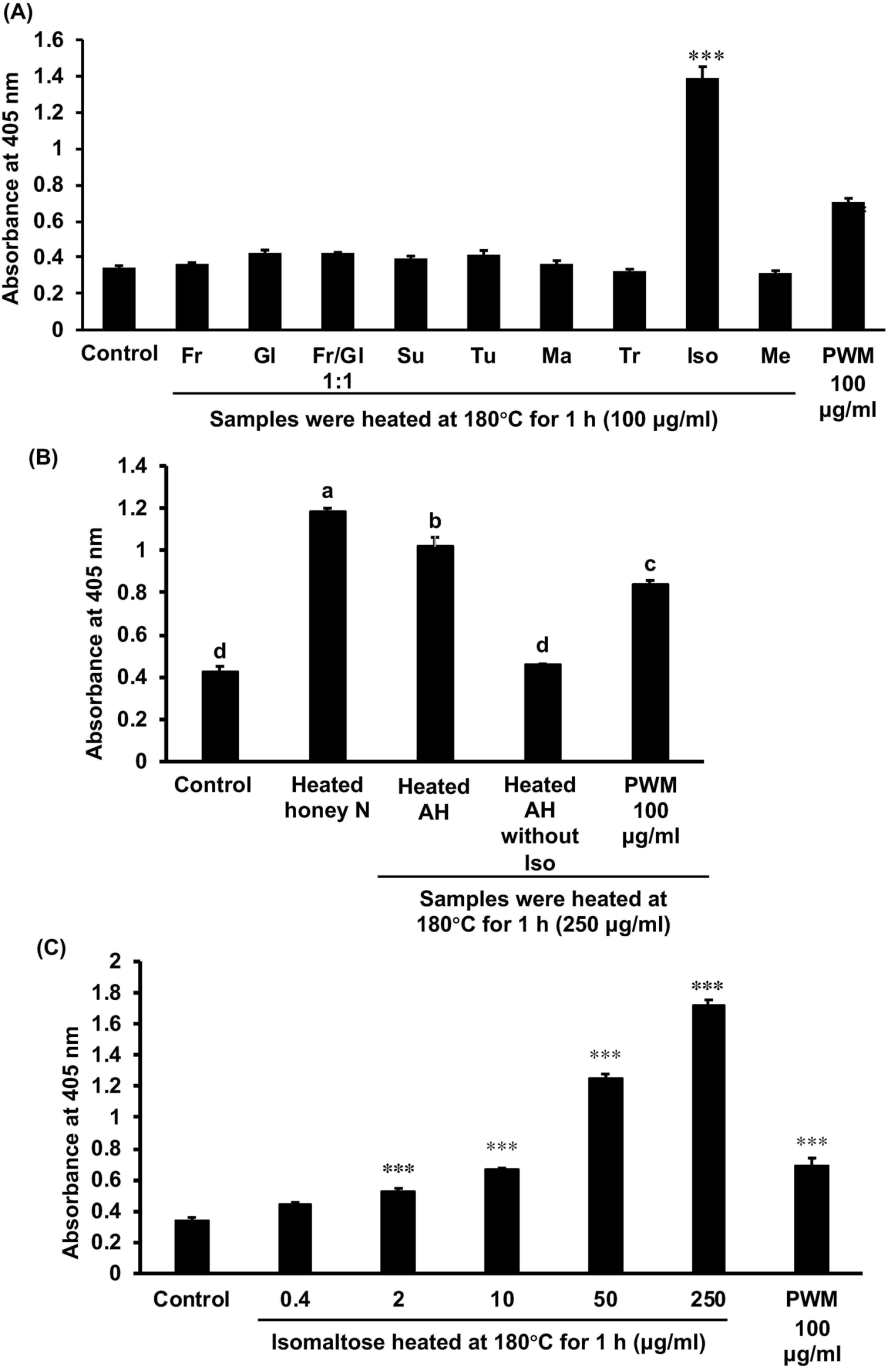
Inducible effect of each sugar or heated artificial honey (AH) heated on the G-CSF secretion in MCE301 cells. (**A**) Each sugar or the mixture of glucose and fructose (1:1), (**B**) AH with or without isomaltose, or (**C**) isomaltose was heated at 180 °C for 1 h. (**B**) AH with or without isomaltose was prepared according to the ratio of sugar contents in honey sample N and the contents of sugars in AH with or without isomaltose shown in Supplementary Table 1. After being heated, the samples were dissolved in the medium, and the cells were incubated for 24 h. The G-CSF levels in the medium were measured by ELISA and the data are shown as the absorbance. (**A**,**C**) ***p < 0.001 against the control group by Dunnet’s multiple *t*-test. (**B**) The different letters over the columns indicate significant treatment differences as determined by Dunnet’s and Dunn's multiple *t*-tests (*p* < 0.05). Data are represented as the mean ± S.E. (*n* = 6). *Fr* fructose, *Gl* glucose, *Su* sucrose, *Tu* sucrose, *Ma* maltose, *Tr* trehalose, *Iso* isomaltose, *Me* melezitose, *PWM* pokeweed mitogen.

Among the sugars evaluated, only heated isomaltose could induce G-CSF secretion significantly when heated at 180 °C for 1 h (Fig. 3A). Since the correlation between the amount of turanose and G-CSF induction was significant (Fig. 1D), we evaluated the effect of heated turanose on G-CSF induction. Heated turanose did not exhibit any effect when the heating duration was 1 h or less, though it had a significant effect when heated at 180 °C for 2 and 4 h. However, this effect was significantly weaker than that of honey sample N heated for 30 min (Fig. 2C). In order to evaluate the contribution of isomaltose to the effect of honey, artificial honey, with or without isomaltose, by mixing single sugar compounds according to the ratio of the content in honey sample N (Supplementary Table 1). Heated artificial honey without isomaltose did not lead to G-CSF induction, although heated artificial honey and honey sample N exhibited did (Fig. 3B). Heated isomaltose significantly induced the G-CSF induction in a dose-dependent manner (Fig. 3C).

### Real-time quantitative reverse transcription polymerase chain reaction (RT-qPCR)

We could not observe any significant induction of the G-CSF mRNA expression when mRNA was collected from the cells treated with heated isomaltose for 3, 6, 12, and 24 h (data not shown). A significant induction of the G-CSF mRNA expression was only observed in the cells treated with heated isomaltose for 1 h. The ratios between the G-CSF mRNA expressions subtracted by the β-actin mRNA expressions to those of the control group were 1.00 ± 0.04 (control), 1.30 ± 0.06 (100 µg/ml of heated isomaltose, *p* < 0.05), and 1.99 ± 0.11 (100 µg/ml of lectin from *Phytolacca americana* (pokeweed mitogen, PWM), *p* < 0.01, Dunnet’s and Dunn’s multiple *t*-tests) (*n* = 6).

### Screening for the isomaltose optimum temperature and heating duration to induce maximal G-CSF secretion

**Figure 4:**
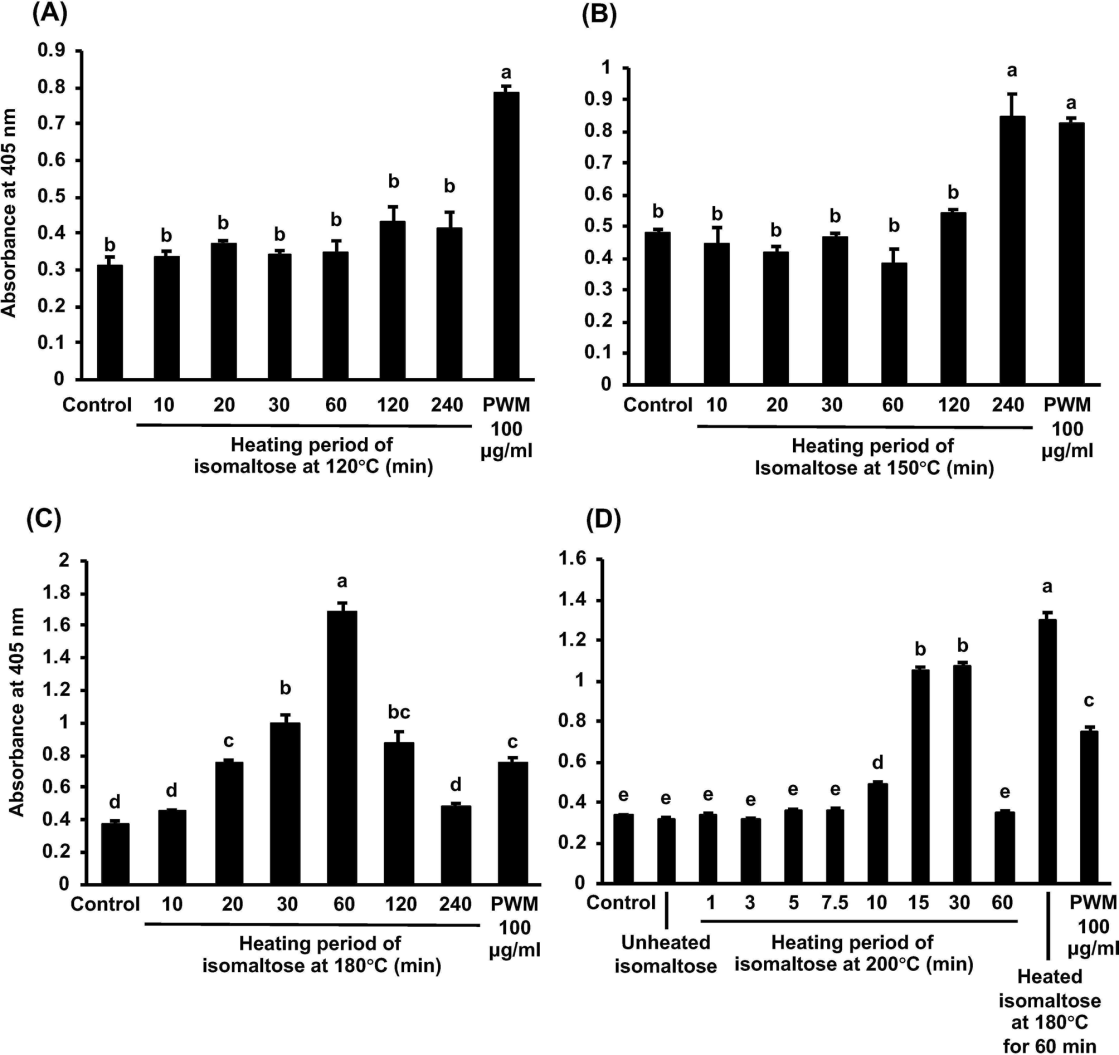
Inducible effect of isomaltose heated at various conditions on the G-CSF secretion in MCE301 cells. After being heated, the samples were dissolved in the medium at 250 µg/ml, and the cells were incubated for 24 h. The G-CSF levels in the medium were measured by ELISA and the data were shown as the absorbance. The different letters over the columns indicate significant treatment differences as determined by Dunnet’s and Dunn’s multiple *t*-tests (*p* < 0.05). Data are represented as the mean ± S.E. [*n* = 3 for (**A**–**C**); *n* = 5 for (**D**)]. *PWM* pokeweed mitogen.

We could not detect any effects in any samples when isomaltose was heated at 120 °C (Fig. 4A). When heated at 150 °C, the G-CSF secretion was only observed after 240 min (Fig. 4B). At 180 °C, the activities were significantly enhanced in a time-dependent manner throughout the first 60 min. However, this effect diminished in a time-dependence after heating for more than 120 min. Importantly, the isomaltose heated for 240 min did not affect the cells (Fig. 4C). At 200 °C, the activities were also significantly increased in a time-dependent manner from 15 to 30 min, and then lost when heated for 60 min (Fig. 4D).

### The characteristics of the heated isomaltose active ingredient

**Figure 5:**
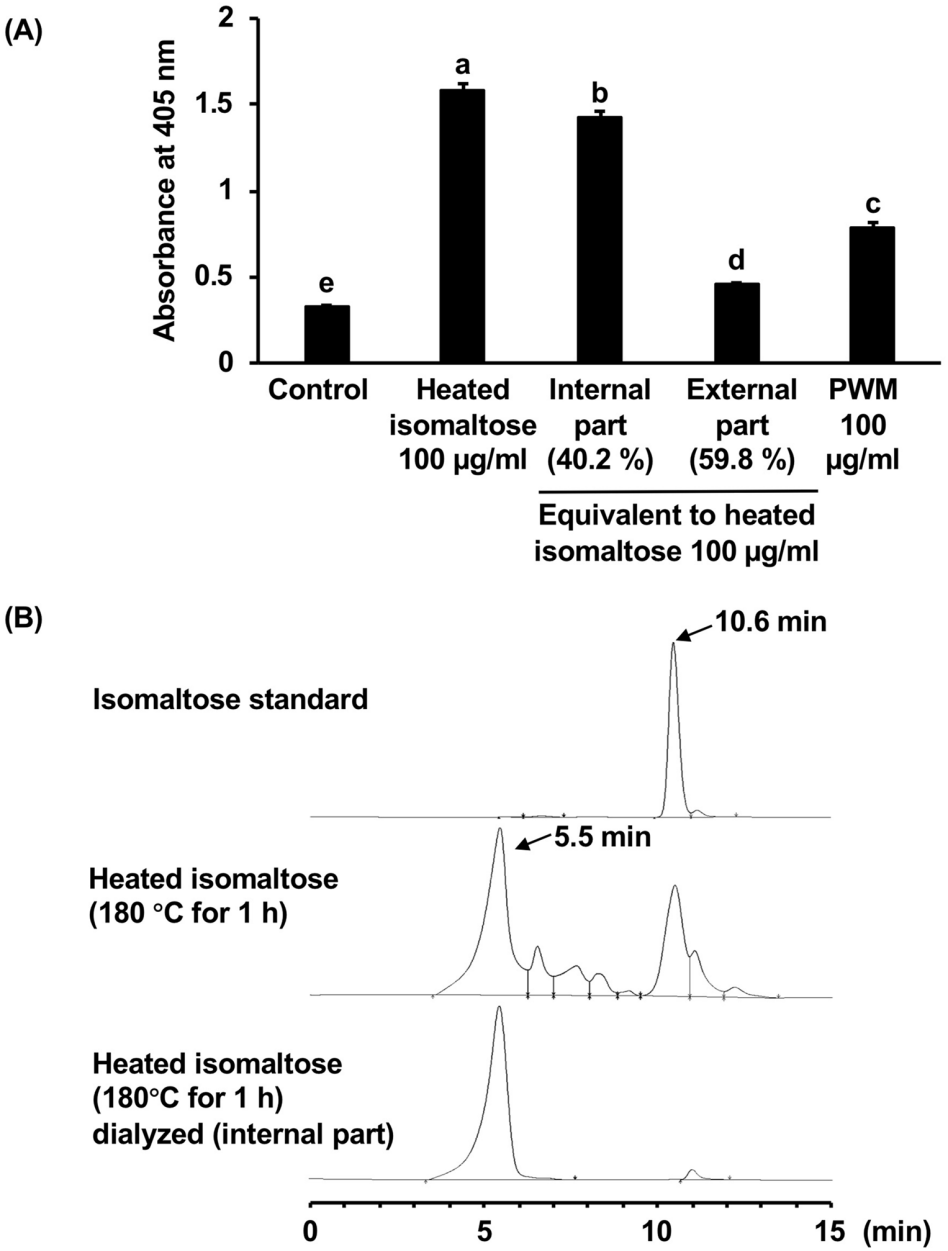
Prediction of the chemical structure of heated isomaltose. (**A**) Isomaltose was heated at 180 °C for 1 h, then partitioned by a dialysis membrane with a molecular weight cutoff value of 12,000–14,000. The samples were dissolved in the medium at an equivalent concentration to the heated isomaltose (100 µg/ml) and the cells were incubated in it for 24 h. The G-CSF levels in the medium were measured by ELISA and the data were shown as the absorbance. The different letters over the columns indicate significant treatment differences as determined by Dunnet’s and Dunn’s multiple *t*-tests (*p* < 0.05). Data are represented as the mean ± S.E. (*n* = 6). (**B**) HPLC chromatogram of isomaltose, heated isomaltose, and its high molecular weight fraction using size exclusion liquid chromatography. HPLC conditions: column, Inertsil WP300 Diol column (250 mm × 4.6 mm i.d., 5 μm, GL sciences, Tokyo, Japan); mobile phase, water, 0.3 ml/min; column temperature, 40 °C; injected volume, 10 μl; detector, refractive index detector RID-20A (Shimadzu). *PWM* pokeweed mitogen.

When dialyzed the heated isomaltose, the active ingredient was mainly transferred into the high-molecular-weight part (Fig. 5A). We analyzed the average molecular weight of this part by size-exclusion HPLC and represented the results of chromatograms, shown in Fig. 5B. Isomaltose was eluted at 10.6 min, and high-molecular-weight compounds appeared after heating. Following the separation through the dialysis membrane and low-molecular-weight ingredients removal, the main single peak was eluted at 5.5 min. The average molecular weight of this product was estimated to be 790 kDa.

Thin-layer chromatography (TLC) analysis revealed that one spot was appeared in the hydrolysates of the high-molecular-weight part by TFA. We isolated this spot, and identified it as glucose by nuclear magnetic resonance (NMR) and mass (MS) analyses.

### G-CSF induction assessment via toll-like receptors (TLRs)

**Figure 6:**
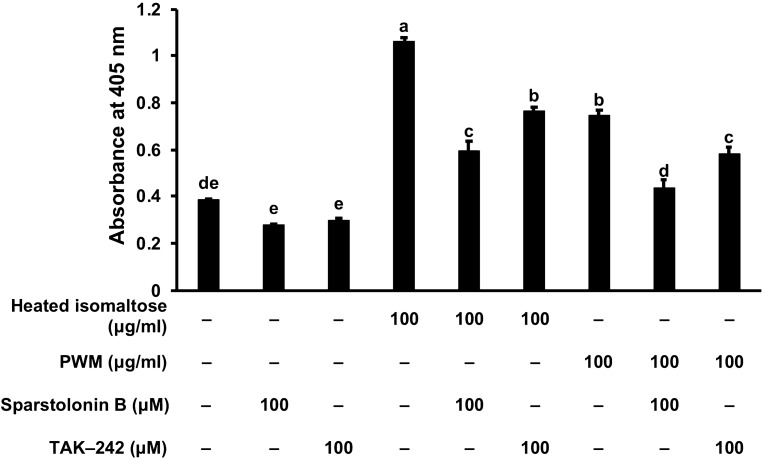
Counteractive effects of sparstolonin B or resatorvid (TAK-242) on the inducible effects of heated isomaltose (180 °C for 1 h) on the G-CSF secretion in MCE301 cells. Cells were pre-treated with sparstolonin B or TAK-242 for 2 h, then co-incubated with heated isomaltose or PWM for 1 h. The G-CSF levels in the medium were measured by ELISA and the data were shown as the absorbance. The different letters over the columns indicate significant treatment differences as determined by Dunnet’s and Dunn’s multiple *t*-tests (*p* < 0.05). Data are represented as the mean ± S.E. (*n* = 5). PWM: pokeweed mitogen.

Since TAK-242 (an inhibitor of TLR4) appeared to be cytotoxic to the cells when treated for 24 h, the pretreatment incubation thus lasted for only 2 h and the induction for 1 h. We observed significant cytotoxicity in the groups pretreated with sparstolonin B for 2 h (approximately 80% of the control) but other groups treated with heated isomaltose and PWM for 24 h with or without the pretreatment with TAK-242 pretreatment for 2 h did not exhibit any significant cytotoxicity (data not shown). The G-CSF secretion-inducing activities of the heated isomaltose and the PWM were significantly inhibited by sparstolonin B (an inhibitor of both TLR2 and TLR4) or TAK-242 (Fig. 6).

## Discussion

Intestinal epithelial cells form a barrier that protects the individuals from the external environment. Previous studies have reported that these cells could advance not only their own proliferation and differentiation, but also regulate the function of immune cells through the induction of cytokines, such as interleukins, tumor necrosis factors, thymic stromal lymphopoietin, and colony-stimulating factors^19,20^. Among these cytokines, G-CSF recruits monocytes and macrophages into the intestinal membrane^21,22^, which is why we believe that the induction of G-CSF secretion from intestinal epithelial cells is implicated in tonifying *qi*.

In the present study, we found that the induction of G-CSF secretion in MCE301 cells by heated honey samples from China tended to be higher than those from Japan, as well as corn syrup products. The major sugars in all the honey samples were fructose and glucose, with minor saccharides varying among products^17,23,24^. Thus, it is likely that their unique profiles contributed to the differences in inducing G-CSF secretion. Then, we evaluated the relationships among sugar contents, the ratio of glucose/fructose, and the induction of G-CSF secretion.

Among honey samples, the contents of each sugar varied depending on the original flowers and producing companies. The glucose content in honey samples purchased in China was significantly lower than that in Japan. The contents of turanose, maltose, and isomaltose in honey samples purchased in China tended to be higher than those in Japan (not statistically significant). Since turanose, maltose, and isomaltose are disaccharides of glucose groups, disaccharides in honey purchased in Japan might more hydrolyze into glucose than those in China. Among honey samples derived from jujube flowers, the contents of these sugars varied substantially, suggesting that the variation of sugars in honey would not depend on the origin of honey but the companies produced. There was no correlation between the induction of G-CSF secretion and the contents of glucose or fructose in honey samples, but the mixture of glucose and fructose did generate the activity when heated to 180 °C for 2 h, and the best ratio of glucose/fructose for the appearance of the activity was of 1:1. This phenomenon was also appeared in sucrose, the disaccharides of glucose and fructose. We found the amount of turanose in honey was significantly correlated with the activity of honey after heating at 180 °C for 30 min. However, turanose did not exhibit the activity in this heating condition but was heated for more than 2 h. For isomaltose, the activity appeared after it was heated to 180 °C for 20 min, and the time- and temperature-dependent manners of isomaltose exhibited were similar to those of honey in our previous study^4^.

According to the results of the correlation analysis and the comparison of heated artificial honey with or without isomaltose, we could conclude that isomaltose in honey is the primary ingredient leading to the induction of G-CSF secretion in MCE301 cells when heated. However, heated artificial honey with isomaltose exhibited 86% of the activity of heated honey sample N (*p* < 0.05), suggesting that ingredients other than isomaltose in honey sample N might partially contribute to the induction of G-CSF. In a previous report, protein–polyphenol–oligosaccaride complexes with a high-molecular-weight appeared when honey was heated^25^, and as such, we inferred that undetected sugars, polyphenols, and proteins contained in honey might be implicated in this finding.

We did not detect any significant induction of G-CSF mRNA expression when cells were treated with heated isomaltose beyond 3 h, although it was detected when cells were treated for 1 h. It is possible that stimulation with heated isomaltose induces G-CSF mRNA expression very quickly but disappears after 3 h. Indeed, media of the cells incubated with heated isomaltose for 1 h contained significantly higher amounts of G-CSF than that for the control (data not shown). Such a response to external stimulation has also appeared among macrophages^26^, indicating that protein synthesis does not always require de novo mRNA synthesis^27,28^. Here, heated isomaltose might increasing the translational efficiency of G-CSF in MCE301 cells.

In the previous reports, peritoneal macrophages and MCE301 cells have been demonstrated to produce G-CSF in response to bacterial endotoxins such as lipopolysaccharide (LPS)^29–31^. LPS is a known agonist to both TLR2 and TLR4^32–34^, and PWM activates B cells via TLR2^35^. In our study, the induction of G-CSF secretion by heated isomaltose or PWM was significantly inhibited by sparstolonin B (an antagonist of both TLR2 and TLR4), though only partially by TAK-242 (inhibitor of TLR4). This suggests that heated isomaltose might enhance G-CSF secretion by activating at least TLR4 signaling pathway. There are several reports that polysaccharides moderate biological activity via TLR2/TLR4 in immune response, as polysaccharides purified from *Acanthopanax koreanum* stimulated B cell activity via TLR2/TLR4^36^, and reishi polysaccharides also induced immunoglobulin production through the same receptors^37^. In downstream signaling pathways from TLR2/TLR4, nuclear factor‐κB, extracellular signal-regulated kinase (ERK)1/2, and mitogen-activated protein kinase have been reported to be involved in the induction of immunostimulatory activity^38,39^, some of which were suppressed by sparstolonin B^40^. In addition, the G-CSF-mediated JAK2/STAT3 pathway played an essential role in hematopoietic function^41,42^. In order to elucidate the detailed mechanism of heated isomaltose on immunostimulatory activity, further investigations examining the downstream signaling pathways are necessary.

We screened the suitable temperature and heating duration required for isomaltose to induce G-CSF secretion, and the greatest effect appeared when isomaltose was heated at 150 °C for 240 min, at 180 °C for 60 min, or at 200 °C for 15–30 min, which was similar to the results of honey and licorice roasted with honey from our previous study^4,13^. We surmise that certain novel active compounds were produced when honey was heated but degraded upon excess heating. The Chinese Pharmacopoeia does not describe the suitable heating temperature and period for honey-processing but does recommend “stir-baking” until crude drugs become yellow to deep yellow and not sticky to the fingers^3^. Usually, the heating temperature on the woks is around 200–350 °C^43^. Heating to 200 °C for 15 min, or at a higher temperature for a shorter period, would be possible in stir-baking. Thus, our findings reveal that this could be a suitable condition for the processing method of stir-baking with honey in TCM.

Novel polysaccharides isolated from the fruiting bodies of *Grifola frondosa* (a polypore mushroom) and the stem of *Dendrobium officinale* (an orchid) were discovered to have an average molecular weight of 27.2 kDa and 534 kDa, respectively, and are immunomodulators^44,45^. Although some other reports describing polysaccharides with immunostimulatory activity isolated from plants have been published previously, no investigations have examined the appearance of immunostimulatory activity from a certain sugar by heating to our knowledge. From the results of our size-exclusion HPLC, the average molecular weight of the active ingredient in isomaltose heated at 180 °C for 60 min was estimated to be 790 kDa. This was similar to the 730 kDa product identified in our previous study^4^. Upon further evaluation, glucose was found to be the only hydrolysate, suggesting that the glucose-groups in isomaltose molecules were oxidatively polymerized. Since maltose, another glucose disaccharide, did not exhibit the appearance of this activity when heated, it is plausible that the *α*(1 → 6) binding motif between two glucose molecules plays an important role in the impact of heated sugars on immunostimulatory activity.

In conclusion, isomaltose was found to be the primary component in honey when heated to oversee the significant induction of G-CSF secretion from intestinal epithelial cells, making it a useful marker ingredient of honey products suitable for application in TCM. Although isomaltose does not exert any pharmacological actions on the immune system, heat processing produces the immunostimulatory activity, with the best conditions being at 180 °C for 60 min or 200 °C for 15–30 min. The average molecular weight of the compound produced was 790 kDa, and this compound induced G-CSF secretion via TLR4. Although further studies, including experimental animal studies and clinical trials, are required, it holds great potential as a therapeutic agent for the patients with immune systems compromised by aging, anticancer drugs, or lifestyle-related diseases.

## Methods

### Materials

Twenty honey and two corn syrup samples were purchased from companies in China and Japan in 2017 (Table 1). Fructose and maltose were purchased from Nacalai Tesque (Kyoto, Japan). Glucose and sucrose were purchased from Fujifilm Wako Pure Chemical Industries (Osaka, Japan). Turanose, trehalose, and isomaltose were purchased from Tokyo Chemical Industry (Tokyo, Japan). Melezitose was purchased from Kanto Chemical (Tokyo, Japan).

### Preparation of the heated samples

Each sample of honey or corn syrup (about 100 mg) was dropped into a glass test tube, and the accurate weight was measured. Tubes without tops were heated at 180 °C for 30 min, and then weighed. Distilled water (10 ml) was added, and the mixture was kept in boiling water for 30 min until the products dissolved. The solutions were lyophilized and weighed (Table 1). The dried products were dissolved in water at a concentration of 100 mg/ml, and stored at − 20 °C until use.

Each sample of fructose, glucose, sucrose, turanose, maltose, trehalose, isomaltose, and melezitose was dissolved in distilled water at a concentration of 100 mg/ml. Artificial honey, with or without isomaltose, by mixing single sugar compounds according to the ratio of the content in honey sample N, and the prescriptions of sugars are shown in Supplementary Table 1. The sugar mixture was dissolved in distilled water at a concentration of 1 g/ml. Each solution (300 μl for the sugar mixture and artificial honey samples, 100 μl for the single sugar samples) was dropped in a glass tube, and heated to 80 °C to evaporate the liquids. Then, the tubes without tops were heated using different protocols (120–200 °C, 0–240 min). The products were then weighed and dissolved in distilled water at 100 mg/ml for the sugar mixture and artificial honey samples, 5 mg/ml for the single sugar samples.

### Analysis of the sugar contents and molecular weights by high-performance liquid chromatography (HPLC)

Seventy microliters of each honey sample solution (100 mg/ml) or the mixed sugar solutions were lyophilized, then dissolved in 100 µl of acetonitrile–water (3/7). After centrifugation at 14,000 × *g* for 5 min, the supernatant was analyzed in the HPLC system (Alliance HPLC; Waters, Milford, MA, USA) with the following parameters: column, Sugar D (4.6 × 250 mm, Nacalai); mobile phase, acetonitrile–water (85/15); flow rate, 1.0 ml/min; column temperature, 40 °C; and detection, refractive index detector (2414 RI detector, Waters). Fructose, glucose, sucrose, turanose, maltose, trehalose, isomaltose, and melezitose were eluted at 11.1, 15.5, 25.7, 27.6, 33.6, 42.1, 43.8, and 64.1 min, respectively. Representative chromatograms are shown in Supplementary Fig. 1. The measurement of the sugar contents was conducted by an external standard method, and the quantitative ranges for each sugar were as 17.5–70.0 µg/ml for fructose and glucose, and 0.11–3.57 µg/ml for the other sugars. Size-exclusion HPLC was performed according to the method described in our previous study^4^.

### Assessment of immunostimulatory activity and the signaling pathways activated by heated sugar

In order to assess immunostimulatory activity, experiments were conducted similarly to our previous investigation^4^. Briefly, murine colonic epithelial MCE301 cells^46^ were cultured in Dulbecco’s modified Eagle medium/Ham’s F-12 (1:1) (DMEM/F-12) medium (Sigma Aldrich, St. Louis, MO, USA) containing 5% fetal bovine serum (FBS; Sigma), 100 units/ml penicillin and 100 µg/ml streptomycin (both from Nacalai). Cells were seeded into 96-well plates at 4 × 10^4^ cells/well, and cultured for 24 h. The cells were then cultured at 39 °C in FBS-free DMEM/F-12 for 3 days. The media were then replaced with those containing samples, and the cells were further cultured for another 24 h at 39 °C. PWM (Sigma) was used as a positive control. In order to gain insight into the signaling pathways activated by heated isomaltose, cells were pretreated for 2 h with sparstolonin B (Sigma), an antagonist of both TLR2 and TLR4, or resatorvid (TAK-242, Medchemexpress, Monmouth Junction, NJ, USA), a TLR4 inhibitor. The cells were then co-incubated with heated isomaltose or PWM for 1 h. The media were transferred to a 96-well plate and evaporated at 38 °C for enzyme-linked immunosorbent assay (ELISA). In order to assess the cytotoxicity, the remained cells were incubated in FBS-free media with MTT (0.5 mg/ml; Sigma) and then were incubated at 37 °C for 1 h. Real-time quantitative reverse transcription polymerase chain reaction (RT-qPCR) was conducted by seeding the cells into 24-well plates at 2 × 10^5^ cells/well, and treated them as mentioned above. Total RNA was extracted from the cells using RNAiso Plus (500 µl/well; Takara Bio, Kusatsu, Japan) and purified according to the manufacturer’s instructions.

### Enzyme-linked immunosorbent assay (ELISA)

The ELISA was conducted according to the methods detailed in our previous study^4,13,47^, although with a slight modification using Can Get Signal Immunostain Immunoreaction Enhancer (Toyobo, Osaka, Japan) to dilute the antibodies. After the media were evaporated, wells were washed with phosphate buffered saline (0.15 M, pH 7.4, PBS), and incubated with Block Ace (undiluted solution, DS Pharma Biomedical, Osaka, Japan) at 25 °C for 2 h. The first antibody (polyclonal anti-G-CSF Picoband Antibody, PB9577, 1:500, Boster Biological Technology, Pleasanton, CA, USA) was then added to the wells and incubated at 25 °C for 2 h. The wells were washed with PBS and incubated with the secondary antibody (anti-goat IgG antibodies conjugated with horseradish peroxidase, 1:2,000, Jackson Immuno Research Laboratories, Inc., West Grove, PA, USA) at 25 °C for 2 h. After washing with PBS again, the ABTS solution (Roche Applied Science, Upper Bavaria, Germany) was added and the absorbance at 405 nm was obtained.

### Real-time quantitative reverse transcription polymerase chain reaction (RT-qPCR)

The real time RT-qPCR was conducted similarly to the method detailed in our previous study^13^. Briefly, RNA (500 ng) was transformed into cDNA using 5 × PrimesScript RT Master Mix (2 µl; Takara), according to the manufacturer’s instructions. RT-qPCR was performed using the SYBR Select Master Mix (Applied Biosystems, CA, USA) and a StepOne Real-Time PCR System (Applied Biosystems). The analysis was carried out using the comparative cycle threshold method (∆∆Ct)^48^. Data were expressed as fold changes of G-CSF/β-actin compared with those of the control.

### Dialysis of heated isomaltose

A 2 ml solution of isomaltose (100 mg/ml) was heated to 80 °C overnight to evaporate the liquids, and then heated at 180 °C for 1 h. About 10 ml of distilled water was added and the sample was kept standing in boiling water for 20 min to dissolve the heated isomaltose. The solution was lyophilized, dissolved in distilled water (50 mg/ml), and dialyzed using a Spectora/Por Dialysis Membrane (cutoff 12,000–14,000, Spectrum Laboratories, Bridgend, UK) for 3 d. Each internal (high-molecular-weight part) or external (low-molecular weight part) solution was collected, evaporated, lyophilized, and then the high-molecular-weight part (37.6 mg) and the low-molecular-weight part (56 mg) were obtained.

### Identification of the hydrolyzed product from heated isomaltose

The high-molecular-weight part (35 mg) obtained from the procedure described above was dissolved in 1.75 ml of 2 M TFA and heated at 100 °C for 3 h. The TFA was evaporated off, and the product was dissolved in water and extracted with ethyl acetate to yield the water layer (11.7 mg) and the ethyl acetate layer (0.48 mg). Thin-layer chromatography (TLC) was conducted using the following conditions: plate, silica gel 60 F_254_, (Merck, Darmstadt, Germany); developing solvent, ethyl acetate/methanol/water (7:2:1); detection, spraying with 0.2% of 1,3-naphthalenediol (Fujifilm) and 10% phosphoric acid in ethanol, then heated at 105 °C for 3 min. A single spot (*Rf* value = 0.23) from the TLC plate spotted with the water layer of the hydrolytic product was collected and analyzed by NMR and MS analyses.

### Statistics

One-way analysis of variance (ANOVA) and Dunnet’s and Dunn’s multiple *t*-test were used to compare multiple data. Student’s *t*-test was used to compare two independent groups. Pearson correlation analysis and regression analysis were carried out to evaluate the relationships between sugar content and the induction of G-CSF secretion in MCE301 cells. Data are expressed as the mean ± standard error (SE), and *p* < 0.05 was considered significant. All analyses were conducted using PASQ Statistics (version 18; SPSS, IBM, Armonk, NY, USA; https://www.spss.com.hk/statistics/).

## Supplementary information

Supplementary Information.
