# Supplementary material for: Honey isomaltose contributes to the induction of granulocyte-colony stimulating factor (G-CSF) secretion in the intestinal epithelial cells following honey heating
Source: Sci Rep. 2020 Sep 16;10:15178. doi: 10.1038/s41598-020-71993-w (PMC7494892; doi:10.1038/s41598-020-71993-w)
Supplement: Supplementary file 1 — Supplementary Information. [file 41598_2020_71993_MOESM1_ESM.docx]

Scientific Reports Supplementary Table and Figure

**Honey isomaltose contributes to the induction of granulocyte-colony stimulating factor (G-CSF) secretion in the intestinal epithelial cells following honey heating**

Xin Xu^1^, Koshi Asai^1^, Daiki Kato^2^, Kan’ichiro Ishiuchi^1^, Kewen Ding^1^, Yoshiaki Tabuchi^3^, Misato Ota^1,2^, Toshiaki Makino^1,^*

^1^Department of Pharmacognosy, Graduate School of Pharmaceutical Sciences, Nagoya City University, 3-1 Tanabe-Dori, Mizuho-ku, Nagoya 467-8603, Japan. ^2^Kuki Sangyo Co. Ltd., 11 Onoe-cho, Yokkaichi, Mie 510-0059, Japan. ^3^Division of Molecular Genetics Research, Life Science Research Center, Toyama University, 2630, Sugitani, Toyama 930-0194, Japan. Correspondence and requests for materials should be addressed to T.M. (email: makino@phar.nagoya-cu.ac.jp)

| **Supplementary Table 1.** Contents of each sugar in artificial honey (AH) samples. | | | | | | | |
| --- | --- | --- | --- | --- | --- | --- | --- |
|  | Fructose | Glucose | Sucrose | Turanose | Maltose | Isomaltose | Total |
| Honey sample N^1)^ | 40.7 | 25.9 | 0.1 | 2.9 | 1.1 | 3.6 | 74.3 |
| AH | 54.8 | 34.9 | 0.1 | 3.9 | 1.5 | 4.8 | 100 |
| AH without isomaltose | 57.5 | 36.7 | 0.1 | 4.1 | 1.6 | – | 100 |

The contents of each sugar in each dried sample was expressed as (w/w) %.

Honey sample N ^1)^ is the same as in Table 2.

**Supplementary Figure 1.** Representative chromatogram of honey samples. (a) Standard compounds. (b) Honey sample N. HPLC conditions were described in Materials section.
